# Recent Advances in DNA Origami-Enabled Optical Biosensors for Multi-Scenario Application

**DOI:** 10.3390/nano14231968

**Published:** 2024-12-07

**Authors:** Ziao Hao, Lijun Kong, Longfei Ruan, Zhengtao Deng

**Affiliations:** State Key Laboratory of Analytical Chemistry for Life Science, National Laboratory of Microstructures, College of Engineering and Applied Sciences, Nanjing University, Nanjing 210023, China

**Keywords:** DNA origami, biosensor, transformation strategy, dynamic response, application

## Abstract

Over the past few years, significant progress has been made in DNA origami technology due to the unrivaled self-assembly properties of DNA molecules. As a highly programmable, addressable, and biocompatible nanomaterial, DNA origami has found widespread applications in biomedicine, such as cell scaffold construction, antimicrobial drug delivery, and supramolecular enzyme assembly. To expand the scope of DNA origami application scenarios, researchers have developed DNA origami structures capable of actively identifying and quantitatively reporting targets. Optical DNA origami biosensors are promising due to their fast-to-use, sensitive, and easy implementation. However, the conversion of DNA origami to optical biosensors is still in its infancy stage, and related strategies have not been systematically summarized, increasing the difficulty of guiding subsequent researchers. Therefore, this review focuses on the universal strategies that endow DNA origami with dynamic responsiveness from both de novo design and current DNA origami modification. Various applications of DNA origami biosensors are also discussed. Additionally, we highlight the advantages of DNA origami biosensors, which offer a single-molecule resolution and high signal-to-noise ratio as an alternative to traditional analytical techniques. We believe that over the next decade, researchers will continue to transform DNA origami into optical biosensors and explore their infinite possible uses.

## 1. Introduction

Over the past four decades, DNA nanotechnology has evolved rapidly, guiding numerous developments since it was first proposed by Seeman et al. [1]. In the early stages, research primarily aimed to create useful and complex DNA nanostructures. A notable example is the DNA junctions, which typically take four-armed DNA blocks (the immobile Holliday junction) that are assembled with other building blocks through sticky ends, resulting in products of 1D or 2D periodic arrays or simple stick cubes [2]. However, two major challenges emerge: (1) the excessive flexibility of branched DNA leads to the low stiffness and poor stability of synthesized structures [3] and (2) any stoichiometric deviation of the DNA blocks causes the product to acquire certain dominant structures of unpredictable size and shape, which severely affect the addressability, making site-specific modifications according to the established protocol nearly impossible [3]. In 2006, Rothemund proposed DNA origami technology using circular single-stranded DNA (ssDNA) from the M13 phage genome as a scaffold. By hybridizing 100–200 short staple strands to specific locations on the scaffold through annealing, the scaffold folds into a desired shape [4]. This technique overcomes the defects of poor shape control and low addressability caused by pre-organizing DNA blocks to construct large DNA nanostructures. On such a basis, stereo-conformational DNA origami, such as hollow tetrahedral, cubic, and barrel structures, was developed by folding the formed planar 2D DNA origami. Additionally, three-dimensional curved DNA origami was developed by introducing cross structures and guided surface formation, such as vases, multi-tooth gears, and spherical wireframes [5,6]. Certain DNA origami structures with cavities have been used to load various biomolecules and nanoparticles, such as antibodies, glucose oxidase (GOx), horseradish peroxidase (HRP), streptavidin, bovine serum albumin (BSA), doxorubicin, gold nanoparticles (AuNPs) and silver nanoparticles (AgNPs) [7,8], enabling controlled release and reaction compartmentalization. Furthermore, Wei et al. [9] explored the single-stranded DNA tiles that assemble complex nanostructures without relying on scaffolds. Similar concepts have been applied to the design of 102 distinct three-dimensional DNA structures [10]. In addition, to fully integrate DNA folding with biological systems, Yan et al. came up with a single-strand origami [11], where ssDNA can be cloned into Escherichia coli for replication and amplification. After a simple heating and cooling process, the ssDNA can effectively self-fold into the designed structure. This approach eliminates the need to synthesize all the short ssDNA strands from scratch, significantly reducing costs.

The Watson–Crick base pairing rules endow DNA origami with excellent addressability, enabling highly reproducible production and the site-specific organization of nanomaterials with high spatial resolution. This allows for the rapid validation and adjustment of DNA origami functions. Through reasonable structure design, solution treatments that inhibit nuclease activity, intrastrand or interstrand chemical modification, the application of anti-degradation coatings, and solid substrate adsorption, DNA origami exhibits good stability in both physiological and material processing environments [12,13]. Due to its addressability and stability [12,13,14,15], DNA origami has been introduced into a wide range of fields, including molecular imaging, cell communication, biosensing, smart drug delivery, photothermal therapy, and nanorobots [16]. Although some applications in vivo are well developed, monitoring the trajectory and target effects of DNA origami in living organisms often requires precise, bulky instruments or long periods of observation [17]. Moreover, in most cases, the in vivo application of DNA origami serves as a therapeutic measure after the host suffers danger, such as delivering chemotherapeutic drugs to targeted tumor cells [18] or transporting bactericide to infected wounds [19], with limited potential for early risk detection. DNA origami-based biosensing offers an intriguing opportunity, as it fully leverages dynamic DNA nanostructures, combining both structural and functional properties. This enhances the ability of DNA origami to participate in recognition, signal transduction, and response. Notably, optical sensors can provide quick and simple reporting, expanding the use of DNA origami from professional, institutional settings like hospitals and laboratories to field environments, including water quality monitoring, epidemic prediction, food security surveillance, and even point-of-care diagnostic tools [20,21,22,23].

So far, studies on converting DNA origami into biosensors remain limited. While some studies have applied the same mechanisms to modify different origami structures, the application range of these designed sensors is still quite narrow [24,25,26]. In this paper, we discuss the potential of transforming DNA origami into general biosensors and explore how DNA origami can explore its own functional properties for sensing rather than merely serving as a static element or auxiliary platform in the sensing process. General strategies for the transformation of DNA origami to biosensors are presented in detail. We also analyze the advantages of DNA origami biosensors relative to conventional optical biosensors, as well as the challenges related to their preparation and practical application. In addition, this paper gives insights into the practical application ideas of DNA origami biosensors from a synthetic biology perspective, such as acting as transducers in artificial light capture systems, environmental pollution monitoring, and the creation of wearable organic fluorescent materials. These fields are poised to benefit from the well-established theoretical and practical experience of DNA origami biosensors in biomedicine.

## 2. Requirements and Challenges in Constructing Optical Sensors with DNA Origami

**Figure 1:**
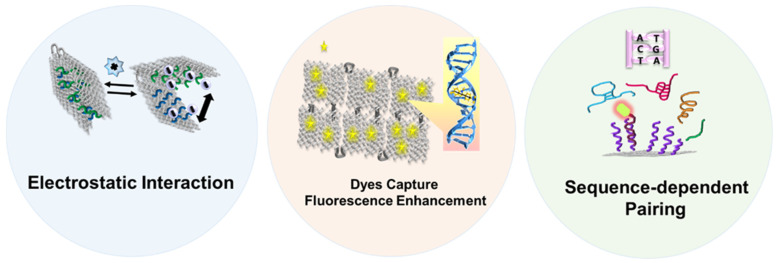
Schematic diagram of three intrinsic response mechanisms of DNA origami [36,37].

A biosensor must contain the following essential elements: a recognition element that interacts with the target molecule or the local microenvironment (pH, temperature, electromagnetic field, etc.) and can distinguish targets from non-targets based on the mode or intensity of action; a transduction element, typically capable of converting molecular binding events or slight environmental changes into physical signals (light, electricity, pressure, etc.); and reading facilities. Human eyes, as the most primitive yet highly effective reader, can directly interpret biological signals, allowing the brain to decode what the recognized object is conveying. In addition, scientists have developed a range of instruments to excite and record signals, such as spectrometers, digital multimeters, oscilloscopes, and various high-resolution microscopes, which provide visual quantitative information. Given these requirements, natural DNA origami still has some limitations in becoming a biosensor, as its inherent recognition and reporting capabilities are finite: (1) its electrostatic interactions are highly sensitive to surrounding cations [24]; (2) it has a specialized ability to capture dyes and enhance fluorescence through groove chimerism and base intercalation [27,28]; and (3) it can screen target DNA/RNA from a nucleic acid mixture through specific base pairing [29] (Figure 1). These restricted properties make it nearly impossible to detect non-fluorescent dyes, non-nucleic acid species, or non-physicochemical parameters.

Nevertheless, with the advancement of nanohybrid oligonucleotide technology, it is now possible to seamlessly fuse natural or artificial receptors (such as transcription factors [30], aptamers [31], etc.), as well as reporting materials (such as fluorescent molecules [32], electroactive substances [33], etc.), with DNA origami. This significantly compensates for the inherent limitations of DNA origami. There are two common approaches: on the one hand, metal-chelating ligands (such as NTA-Ni), biomimetic receptors (such as biotin), or covalent cross-linking groups (such as Sulfo-SMCC) can be attached to the terminals of short ssDNA strands to make the scaffold folded. This allows molecules, such as antibodies or gold nanoparticles, carrying affinity peptide tags, streptavidin, or specific reaction groups, to be easily fixed at the modified ends of the ssDNA, followed by precise assembly using a “one-pot method” [34]. This approach enables DNA origami to acquire additional recognition or reporting capabilities. On the other hand, many biomolecules cannot tolerate the “excessive temperature changes” during origami formation, leading to denaturation or deactivation. In such cases, a post-modification strategy should be adopted, where DNA origami is first synthesized, and then ssDNA “overhangs”, extending from the origami surface and are hybridized to anchor specific molecules at the desired sites [35]. While this approach requires additional oligonucleotides, it effectively protects the introduced molecules and avoids any possible damage to the inherent skeleton.

Finally, an ideal DNA origami biosensor should be compatible with existing reading devices. Based on current research, optical detection platforms appear to be the most promising. This is because the highly complex structure of origami, along with its strongly negatively charged DNA backbone, creates a significant electron transfer barrier, making DNA origami less suitable for electrochemical sensors, as demonstrated by previous studies with aptamer-modified electrodes [38]. Compared to other single-molecule sensors in solution, DNA origami has a visible structure that can be well-characterized using atomic force microscopy or confocal microscopy. Its large surface area also enables the integration of numerous optical elements simultaneously. As a result, with sufficient advancement, even handheld spectrometers or smartphone camera systems could be used to capture and interpret the signals.

## 3. Different Dynamic Response Strategies of DNA Origami-Based Sensors

DNA origami biosensors with light-based signals developed over the past decade can generally be grouped into two categories based on target molecule binding events. Similar to the well-known immunoassay technique “ELISA”, the most significant distinction between these two groups lies in competitive versus non-competitive binding. However, they are not exactly the same as ELISA, as the competing molecules can belong to entirely different species, and the signal transduction methods are not limited to enzyme-labeled antibodies.

### 3.1. Competitive Binding-Based Sensing Scheme

#### 3.1.1. Toehold-Mediated Strand Displacement

**Figure 2:**
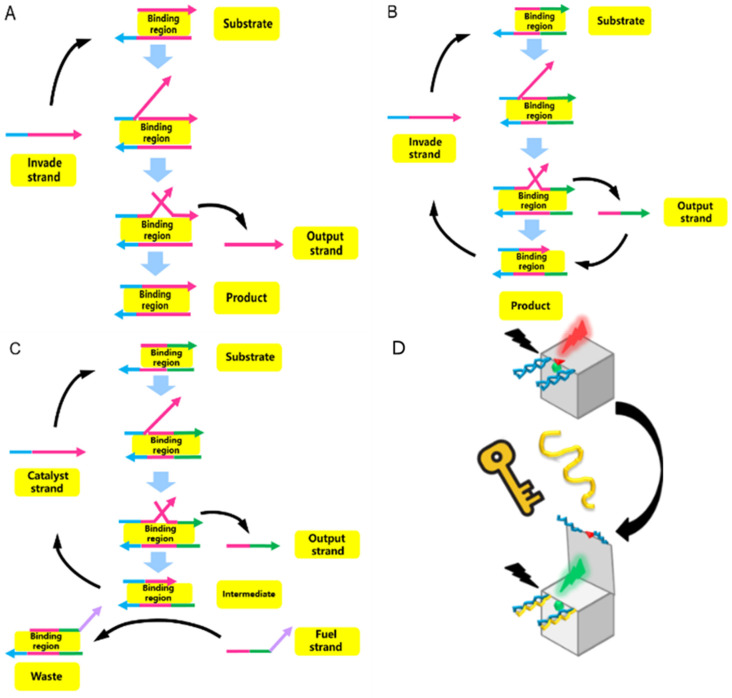
Three common TMSD reactions and a competitive sensing scheme based on TMSD. (**A**) Irreversible TMSD without amplification reaction [39]. (**B**) Reversible TMSD without amplification reaction [39]. (**C**) Catalyst strand-mediated TMSD with amplification reaction [39]. Except for the pink segment, other colored arrows represent the toehold domain. (**D**) Box-like DNA origami sensor using irreversible TMSD without amplification reaction; when the “key” strand was input, FRET would be disrupted [40].

Toehold-mediated strand displacement (TMSD) is a model describing the competition between two forms of ssDNA (‘complementary strand’ means their sequence is partially overlapped) that complement the same substrate strand; ultimately, only the ssDNA that forms the low-energy duplex structure can be left, while the ssDNA with the high-energy duplex structure is expelled. At the microscopic level, toehold association, branch migration, and initial complementary strand dissociation occur successively [39]. The competing ssDNA is usually called the “invade strand” or “fuel strand”. According to Tang et al., if the “invade strand” can completely hybridize with the substrate strand, irreversible displacement (toehold displacement reaction, TD reaction) will be brought about (Figure 2A) [39]. This means that the “expelled strand” can no longer interfere with the formed stable duplex, even if it contains sequences complementary to the substrate strand; if the “invade strand” is only hybridized with part of the substrate strand, the final duplex product still remains a “toehold domain”, and the “expelled strand” can compete again (toehold exchange reaction, TE reaction) (Figure 2B) through this new “toehold domain”, and the outcome of this reaction depends on the forward and backward reaction kinetic constant [39]. This model is one of the most promising strategies for converting DNA origami into a biosensor, as it is fully compatible with any nucleic acid structure in origami.

Andersen et al. constructed an addressable three-dimensional DNA box using 59 staple strands to connect six rectangular pieces of DNA, demonstrating that this complex structure can be manipulated by external DNA inputs. They placed a pair of DNA locks on the lid of the DNA box. Each lock was formed by the hybridization of two complementary ssDNA strands, which extended separately from the front and top surfaces, leaving sticky ends as “toeholds” [40]. A fluorescence resonance energy transfer (FRET) dye pair was positioned between the two locks. When the “key” ssDNA was introduced, the TD reaction was triggered, causing the box lid to open and the FRET signal to weaken (Figure 2D). This sensing mechanism is highly specific and operates like a logic gate: the lid remains closed if irrelevant, ssDNA is used as the “key”, and only when two “key” ssDNAs are added simultaneously does the lid open (AND gate) leading to a complete reduction in the FRET signal. The authors also pointed out that the lid could be designed to remain closed when a specific signal is introduced (NOT gate).

In another scenario, the top and bottom covers could be opened by different “key” ssDNAs (OR gate). Unfortunately, once the DNA box was opened, it could not be closed again due to the fully complementary lock–key system and the strong electrostatic repulsion between the lid and the sides. Zadegan et al. modified the DNA origami box by inserting two locks with stem-loop structures at the edge between the lid and the side, allowing the “key” ssDNA to bind to the toehold domain within the loop and subsequently open the lock stem through strand displacement [41]. To close the DNA origami box again, they designed an 8 nt toehold domain on the 5′ end of the “key” ssDNA, which could react with a “closing key” ssDNA through an inverse TD reaction, effectively shutting the lid. This on–off process could be repeated up to three times, but the “key” and “closing key” must be alternated. Additionally, they designed the “key” strand to match breast cancer-specific biomarkers miR223 and miR30c, giving the DNA origami box the potential to deliver drugs to breast cancer cells.

TMSD has also been applied to study molecular interactions at the single base-pair level. Mathur et al. reported a dumbbell-type molecular interrogating device that controlled the movement of the nucleic acid ring by the user-defined molecular force domain, which could determine whether there were soluble ssDNA virus genome analogs in the solution based on different molecular force configurations when the signal appeared [42]. Three force modes were set up between the ring and the frame at both ends: (1) the pushing force generated by randomly coiled ssDNA hybridization into duplex; (2) the pulling force caused by the collapse of stretched ssDNA when hybridized into duplex; (3) the pushing force generated by the hybridization of a randomly coiled single-stranded ring domain with complementary oligonucleotides. Their different combinations caused the FRET dye pairs that were labeled on the ring and frame to separate, resulting in a signal. To associate the FRET signal with soluble ssDNA viral genome analogs, a toehold-containing staple strand complementary to five Ebola genome sequence elements was extended from the central mobile nucleic acid ring, while the complementary strand extended from the frame. In the absence of viral ssDNA targets, the adhesion between the ring and the frame was strengthened by complementary duplex formation, and FRET changes could only be induced by applying two strong forces of (1) and (3) simultaneously. In contrast, if viral ssDNA analogs were present, they would displace the complementary strands extending from the frame under the guidance of the toehold domain, breaking the ring–frame connection, increasing ring mobility, and thus altering FRET with only (1) or a combination of two weaker forces: (1) and (2).

Similarly, a rhombic DNA nano-origami actuator achieved structure switching controlled by miR-210, which is a biomarker of hypoxic stress in human tumors through TMSD [43]. During DNA origami folding, a pair of staple strands with target recognition sequences were added to the upper and lower corners of the rhombus, and a short ssDNA complementary to the two staple strands was used as a lock to keep the four arms of the rhombus close together. Since the 6-FAM and BHQ-1 tags on the upper and lower adjacent arms were within the Förster radius, no significant fluorescence signal was detected at that time. When miR-210 triggered strand displacement along the 12-base toehold, the lock strand lost its constraint on the four arms of the rhombus, and the origami unfolded due to strong internal electrostatic repulsion, causing 6-FAM to separate from BHQ-1, resulting in the emission of fluorescence. Experimental results indicated that 15 times the lock strand equivalent of miR-210 was required to produce the strongest signal. Additionally, the opening angle of the rhombic origami did not follow a normal distribution, raising concerns about the suitability of this biosensor for biological cancer reporting.

TMSD has demonstrated its versatility in transforming DNA origami into biosensors that detect non-nucleic acid species. Some studies have adapted TMSD by designing non-nucleic acid targets as “invade strands” that compete with complementary strands for aptamer substrates. Heddle et al. reported a DNA nanobox for detecting plasmodium falciparum lactate dehydrogenase (PfLDH). They showed how incorporating PfLDH aptamers into DNA origami by simply extending a pair of staple strands on the lid did not affect the specific binding ability of the PfLDH aptamers [44]. When the optimal complementary strand hybridized with the aptamer, the DNA nanobox remained stably closed in the absence of PfLDH. However, when PfLDH was present, it induced the aptamer to form a hairpin-shaped “aptamer-PfLDH” complex, displacing the complementary strand and causing the lid to open. Similarly, Gao et al. used FAM-labeled ATP aptamers that were bound to complementary strands hybridized with triangular DNA origami-extended staples. ATP induced the dissociation of the fluorescent aptamers, forming a small aptamer–ATP complex and a larger origami–complementary strand complex, creating a sensor that identified signals through centrifugal ultrafiltration [45]. However, these designs are more complex than nucleic acid-triggered TMSD, as the aptamer follows different binding pathways to the target [46]. In the case that it forms a duplex with the complementary strand, the target may lose the opportunity for “conformational selection”. Moreover, key bases involved in binding are often paired with the complementary strand; otherwise, the target will not be able to thermodynamically “expel” the ssDNA complementary to the aptamer [47]. Researchers often need to identify available complementary ssDNA step by step from one end of the aptamer [48] or use molecular docking software to predict key binding sites and selectively design which bases should be occupied by complementary ssDNA. This increases both the cost and risk of the design process.

Multiplexing detection can also be achieved under the support of TMSD in DNA origami. Andersen et al. designed a DNA origami beacon with multi-fluorophores positioned on two rectangular panels and different sensor modules (also named lock) at the top consisting of distinct single-stranded toeholds that could target the oligonucleotide key connected between two panels. Once there was a key input, the two panels would partially open. If all locks were opened by different keys, the distance between two panels became the maximum, so the FRET signal significantly decreased. Therefore, it provides a way to recognize what kind of targets exist in samples according to the FRET decrease degree [32].

#### 3.1.2. Fluorescent Dye–Ligand Displacement

**Figure 3:**
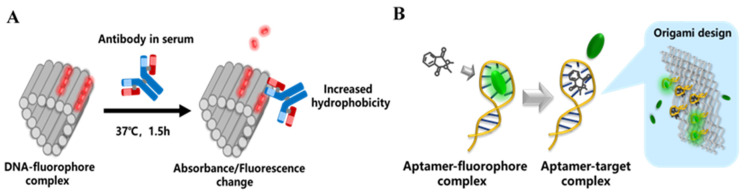
Competitive sensing scheme based on ligand displacement. (**A**) A DNA origami sensor used for autoimmune disease-specific antibodies sensitive detection [27]. (**B**) A fluorescent dye displacement sensor for phthalate detection. This scheme has the potential to be converted into DNA origami sensors [28].

Most nucleic acid fluorescent dyes are quenched by aqueous media, but when they enter the hydrophobic environment of base stacking or the double-stranded DNA (dsDNA) groove, the fluorescence emission of the aromatic core is strongly enhanced [27]. Interestingly, several research groups have reported that antibodies or aromatic contaminants follow a similar mechanism to small molecule fluorophore binding to DNA. These studies have shown that these “non-fluorescent dye species” can displace original dye molecules in DNA–fluorophore complexes [27,28]. For instance, systemic lupus erythematosus produces a large number of anti-DNA antibodies that attack patients’ DNA, targeting specific sequences through hydrogen bonds at the edges of bases and conserved nucleotides. If dyes are pre-bound to the DNA minor groove, some dissociate due to the disruptive effects of the antibodies, while others embed into the hydrophobic epitopes of the antibodies, emitting stronger fluorescence (Figure 3A). This mechanism allows DNA–dye complexes to indicate the presence of antibodies specific to autoimmune diseases. However, using short dsDNA produced a weak signal, while employing larger dsDNA antigens, such as in DNA origami, yielded satisfactory results. This origami has comparable specificity to the gold standard detection scheme “ELISA” used in systemic lupus (81–93% and 80–94% respectively), with less sample volume (2 µL) and a shorter assay time (1.5 h) than ELISA (100 µL and 6.5 h). Its sensitivity is 10-fold higher, and cumbersome steps like pre-coated ELISA plates and washing are omitted. Kim et al. observed that both the intercalating fluorescent dye Popo_3_ and phthalates reduced the positive circular dichroic band at 280 nm of an aptamer (single-stranded DNA), and the presence of phthalates hindered the stable binding of DNA-Popo_3_, resulting in a continuous reduction in fluorescence emissions over 30 min (Figure 3B). Based on this, they designed a biosensor for detecting phthalic acid esters (PAEs) in plastic products [28]. This sensing pattern can be easily replicated into larger structures of DNA by incorporating PAE aptamers into the DNA origami skeleton or choosing other DNA that can bind to Popo_3_ to make origami.

Since nucleic acid dyes can non-covalently bind to DNA and exhibit no biological activity, DNA origami biosensors based on ligand displacement can be synthesized through a “one-pot reaction”. Domljanovic et al. employed an isothermal assembly method at 60 °C, effectively preventing the degradation of common nucleic acid dyes, such as Eva Green and thiazole orange (TO) [27]. However, it is important to note that non-covalent modification using dye molecules can easily lead to DNA structural distortions, which may affect the stability of the biosensors and their specificity toward the target [49].

### 3.2. Non-Competitive Binding-Based Sensing Scheme

#### 3.2.1. Intrinsic Conformational Transformation

**Figure 4:**
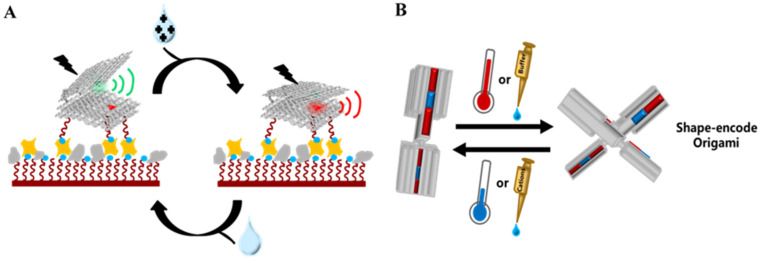
Sensing scheme with self-driven conformational switching capability. (**A**) DNA origami charge-dependent self-driven behavior regulated by cation ion concentration [24]. The cation can shield the electrostatic repulsion between phosphate backbones of short overhangs, prompting hinge arms to tend to “close”. (**B**) The balance between nucleobase stacking and electrostatic interaction dominates the conformational switching of DNA origami, which can continuously sense sensitive parameters in the solution [25].

Since DNA origami biosensors that rely on strand displacement or dye premodification inevitably require external input components, they are not suitable for non-invasive detection. Additionally, these input components must undergo a diffusion search process before interacting with the biosensor [24]. Complex sample environments (e.g., multilayer structures, porous structures) can further hinder signal generation, ultimately preventing signal feedback from occurring on faster timescales. In response, scientists have attempted to develop repeatable actuation and high-time-resolution biosensors based on DNA origami’s response to in-situ physical stimuli. DNA’s charge-dependent, self-driven behaviors significantly influence intramolecular or intermolecular interactions. As is well known, ssDNA molecules are amorphous due to the large number of negative charges carried by the phosphate groups of adjacent nucleotides, which prevents stable arrangement within the chain [50]. After the addition of Mg^2+^/Ca^2+^, ssDNA often forms a well-defined secondary structure due to the screening effect on the phosphate groups’ negative charges [51], which is a feature already utilized in DNA origami scaffolds. Moreover, because DNA polymers are highly negatively charged, they tend to disperse rather than aggregate in solutions [25]. Based on this, Marras et al. reported a cation-activated DNA hinge arm nanodevice capable of accomplishing repeated sub-second, open–close functions [24]. Each arm consisted of a rectangular bundle containing 18 dsDNA helices, together with a specific number of ssDNA staple overhangs retained on the inner surface, and the overhangs at mirror positions on these two opposite surfaces complemented each other. While the complementary sequence on each overhang was too short to stably hybridize, when all short overhangs on both surfaces collectively hybridized, they exhibited a sufficient affinity to bring the hinge arms together. Electrostatic repulsion between phosphate backbones was the primary cause of instability in short overhang hybridization. However, when cations neutralized the negative charges on the phosphate backbones, the stability of the hybridization increased, favoring collective hybridization. Conversely, when cation concentration decreased, the short overhangs rapidly dissociated, leaving the hinge arm open (Figure 4A). The results showed that monovalent, divalent, and trivalent cations in the solution could induce a sensor response, with sensitivity increasing alongside the ionic valence charge. Since this method does not require exogenous DNA binding or the displacement of DNA strands, the DNA origami biosensor demonstrated a reaction time four orders of magnitude faster than the most commonly used TMSD scheme.

The binding of natural substrate pre-transfer RNA (tRNA) to ribonuclease P (RNase P) provides researchers with another method for achieving self-driven DNA origami. The binding pocket of RNase P perfectly fits the receptor stem and T-loop of tRNA geometrically, allowing tRNA to precisely locate the substrate binding region of RNase P, forming stable nucleobase stacking at the shape–complementary interface. Finally, RNase P cleaves the 5′ leader strand of tRNA, producing mature tRNA [52,53]. This process does not involve the complex base pairing typically associated with sequence complementarity. Some studies have confirmed that if different single-layer DNA structures are complementary in their edge shape, they can self-assemble into larger two-dimensional complexes through blunt-end stacking [54]. Gerling et al. demonstrated how short-range nucleobase stacking guides the assembly of three-dimensional DNA origami. They proposed that the balance between stacking bond attraction and electrostatic repulsion at the interface of DNA modules governs the assembly conformation. This balance can be finely tuned by adjusting the concentration of cations or the temperature of the solution, thus enabling three-dimensional DNA origami reconstruction [25]. Specifically, they manufactured blocks with protruding blunt-end double-helix DNA and counterparts with recessed blunt-end double-helix DNA, some of which fit into the grooves of other blocks through surface protrusions, forming a DNA origami “switch”. As the cation concentration increased, electrostatic repulsion at the interface weakened, shortening the average time that the switch remained in the “on” state and shifting the balance toward the “off” state. When a blank buffer was subsequently added, the dilution effect reduced the cations, neutralizing the negative interface charge and inclining the switch toward the “on” state. Temperature regulation was established based on cation concentration, with more DNA origami switches exhibiting the “off” conformation at lower temperatures, while the opposite occurred at higher temperatures (Figure 4B). Remarkably, after 1020 transitions between 25 °C and 50 °C, the origami retained robust reversibility without any structural or functional degradation. This scheme expands the diversity of DNA–origami biosensors sensitive to solution parameters as it programs DNA entirely based on shape complementation. Under the influence of cations, self-complementary monomers can display different structures ranging from nanometers to microns, thereby improving the single-molecule sensing resolution. When solution parameters fluctuate, shape-coded DNA origami manifests continuous intermediate conformations, overcoming the irreversibility of “0–1” binary sensing and reducing the binary signal intensity’s heavy dependence on the number of sensors.

#### 3.2.2. Complementary Strand Hybridization to Unbound ssDNA

In cells, crowded environments (300–400 mg/mL macromolecules) can alter the binding or catalytic activity of biomacromolecules to substrates [55]. For example, phosphoglycerate kinase can close the two subunits that separately bind ADP and diphosphoglycerate without hinge bending in an environment filled with the cell simulation material Ficoll 70, which strongly promotes ATP production [56]. Halpin et al. demonstrated that the heat shock protein Hsp90 occupies more volume in its open conformation and is entropically unstable, while PEG crowding reduces the energy barrier for Hsp90 to transition from the open to the active closed state, thereby activating ATPase [55]. These processes suggest that molecular crowding can induce metastable systems to undergo structural switching. Inspired by this, Hudoba et al. synthesized a DNA origami bucket (named Nanodyn) connected by five “unconstrained” scaffolds, which allowed for spontaneous switching between two stable states at low energy [57]. They anchored Nanodyn to a quartz slide containing biotinylated PEG via streptavidin. When Nanodyn was in the “open” conformation, its extra excluded volume became sensitive to the crowding agent PEG on the slide surface, reducing the entropy of the perturbed PEG molecules and driving the two DNA buckets back to a vertical distribution. At this point, all ssDNA linkers on the scaffolds between the buckets are paired, allowing Nanodyn to remain longer in the “closed” conformation. Furthermore, increasing the concentration of the crowding agent PEG linearly increased the free energy difference (ΔΔGPEG) of Nanodyn between the “on” and “off” states, where ΔΔGPEG was considered a measure of the dissipative force. Dissipative forces affect a range of biological processes, such as the formation of cytoskeletal bundles, DNA, and DNA enzyme binding [57]. As a result, Nanodyn was highlighted as a highly sensitive sensor for detecting important cellular processes since DNA origami can be easily electrotransfected into cells [57].

**Figure 5:**
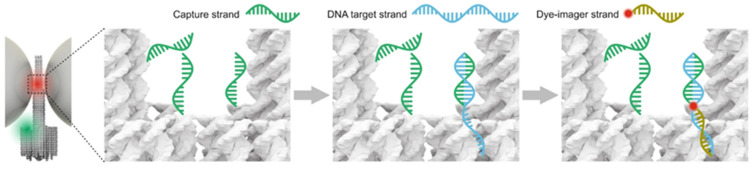
Sensing scheme based on simple complementary strand hybridization [58]. After two rounds of base pairing, the fluorophore is fixed to the plasmonic hotspot, indicating the successful detection of target sequences. Copyright 2021 Nature Communications.

Complementary strand hybridization provides a shortcut to the transition from DNA origami to biosensors. It is worth emphasizing that, unlike TMSD, base pairing does not involve competition for the same ssDNA substrate because ssDNA occupies the unpaired nucleic acid that is free on the origami, or the regions of ssDNA responsible for recognizing, transducing, and reporting binding are spatially separated [43,58]. The addressable capability of DNA origami allows the design of plasma hotspots with customized gaps near the target binding region, thereby enhancing the signal excited by target binding. Trofymchuk et al. used “tower” DNA origami with two pillars to precisely organize silver nanoparticles (AgNPs) that were modified with mercaptylated polythymonucleotides, and these AgNPs bound to the polyadenine nucleotides sticking out of the pillars [58]. In this configuration, a “patio”-like gap was created between the pillars and the AgNPs, which was covered by the surface plasmon of the AgNPs, forming a “hotspot” with an enhanced electromagnetic field. They placed three 17 nt long capture strands into the hotspot and used them to hybridize with the 17 nt specific DNA of antibiotic-resistant Klebsiella, and then the remaining 17 nt specific DNA of antibiotic-resistant Klebsiella was paired with an Alexa Fluor 647-labeled imaging strand (Figure 5). The fluorophore was successfully assembled at the hotspot. The DNA origami biosensor incorporating the hotspot showed a 461-fold stronger signal than a sensor directly performing single-molecule fluorescence detection. Additionally, fluorescence could be easily captured using a portable smartphone monochrome camera with a commercial lens filter, making this approach suitable for point-of-care diagnostic devices.

As mentioned in the previous example, the loose ssDNA coil increases in length and stiffness once hybridized with a complementary strand. Ke et al. utilized this property to design a rhombus-shaped DNA origami scaffold with adjustable distances [43]. They connected the two left arms of the rhombus with two unpaired single-stranded scaffolds, leaving two short types of ssDNA protruding in the symmetrical area on the two right arms to hold the target cargo. When adding a “strut-locking” strand, a rigid double helix formed between the upper and lower left arms. As the strand length increased, the rhombus scaffold gradually stretched open, pulling the right arm in a “mirror” movement corresponding to the left arm. Two split eGFPs (enhanced green fluorescent proteins) were used to verify the dynamic response of the biosensor. When the “strut-locking” strand was fully paired with the two single-stranded scaffolds, the eGFP remained split, emitting only weak fluorescence. However, when the “strut-locking” strand was paired with only a quarter of the single-stranded scaffold, the two eGFP fragments were effectively brought together, resulting in significantly enhanced emission at 524 nm. Similarly, they proposed that the origami biosensor could convert an input into a specific functional output of biomolecules, such as pyruvate kinase and the oxygen-carrying protein hemoglobin.

Meanwhile, base pairing also has the ability to shorten the distance between fixed points. Torelli et al. used an ssDNA probe to connect two distal ends of circular DNA origami, one of which was located on an open flap [59]. Target ssDNA hybridization caused the probe to lose its ability to support the internal tension, reducing the distance between the two ends and thereby opening the flap. They applied this design to cylindrical DNA origami by linking the flap tip to the origami surface with a 120nt ssDNA probe and placing inactive DNA enzymes within the flap. When the external nucleic acid target (such as TMV (tobacco mosaic virus), TSWV (tomato spotted wilt virus), or IYSV (iris yellow spot virus), which are the nucleocapsid genes of several viruses) hybridized with the probe, the flap was pulled apart. This allowed the DNA cargo inside to be assembled into horseradish peroxidase-like G-quadruplex structures by hemin induction, which catalyzed subsequent colorimetric reactions or chemiluminescence. This novel approach enables any DNA origami with a cavity to function as a non-externally excited optical biosensor simply by removing certain staple strands to create a movable surface.

It is difficult to denaturate and regenerate DNA origami biosensors in normal solution environments. In order to restore the available hybridization sites on ssDNA, the separation of sensor–target complexes, dehybridization, and the purification of the sensor itself before reannealing should be considered, which increases the experimental cost. Studying the alternating process of base pairing and dissociation can simplify sensor use. To this end, a research group has explored the continuous response process triggered by base pairing to provide complex information about the two-dimensional origami surface [60]. They fully exploited the tetramerization of streptavidin molecules, connecting each subunit to biotinylated ssDNA and assembling a “molecular spider” with streptavidin as its core and ssDNA as its legs. Three of the legs were 8–17 DNA enzymes capable of binding to a specific oligodeoxynucleotide substrate and cleaving it at the ribose base into two products with low affinity for the enzyme. Once the substrate was cleaved, the 8–17 DNase dissociated and searched for a new substrate nearby, repeating this process. This “molecular spider” had global search capabilities. If it encountered a cleaved product while walking, it would stay briefly due to the higher dissociation constant until it bound to the correct substrate. By setting up “orbits” made of substrates on the origami surface, the “molecular spider” could move directionally and generate defined optical patterns at a macro level. Although this is a proof-of-concept, it offers insight into creating DNA origami biosensors with high-information storage through “base pairing”. By using just one type of fluorophore to mark the unique paths assigned to different targets on DNA origami, nearly infinite multiplexing capabilities could be achieved.

#### 3.2.3. Allosteric Molecule-Driven Structure Change

**Figure 6:**
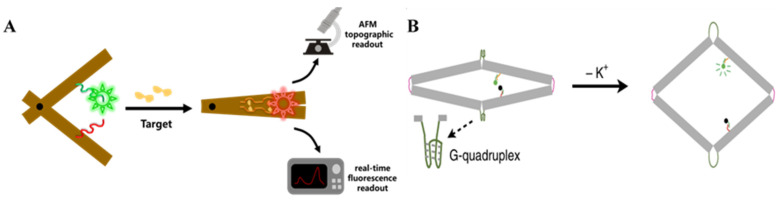
The molecular allosteric effect makes DNA origami acquire an active response capability. (**A**) A DNA origami clamp built from split ATP aptamers. Origami amplifies the target-triggered dimerization process [62]. (**B**) A rhomb-shaped DNA origami biosensor activated by buffer composition. The G-quadruplex DNA structure sensitive to K^+^ remotely controls the signaling unit through intramolecular folding [50]. Copyright 2016 Nature Communications.

Fusing biomolecules with allosteric effects on DNA origami can quickly confer DNA origami with the ability to actively identify targets, thereby amplifying tiny changes at the molecular interface to become instrumentally detectable. Additionally, for allosteric molecules that require monomer heterologous (or homologous) polymerization, DNA origami provides long-range distance variations, significantly reducing false-positive signals in the absence of a target [61]. Walter et al. constructed a pair of split ATP aptamers into clamp-like DNA origami by extending and modifying specific staple strands on two arms that rotated around the same pivot, with each arm linked to one split aptamer stem [62]. When ATP was present in the solution, the two aptamer fragments repolymerized into a complete structure, driving the DNA origami arms to close. At this point, the cyanine-styrene fluorescent dye labeled at the split aptamer end underwent resonance energy transfer (RET), converting the original green fluorescence (observed when the arms were open) to red fluorescence, similar to a “traffic light” transformation (Figure 6A). This design successfully amplified the dimerization of the aptamer upon the binding of small molecules at microscopic levels. Using a similar idea, Xiong et al. designed DNA tweezers that could simultaneously identify aflatoxin and ocher atoxin, except that they hybridized the DNA arm with the complete aptamer [63]. And the target molecule induced the aptamer to fold, followed by its dissociation from the DNA tweezer. As a result, the DNA arms, previously restricted by the aptamer, opened, and the quenched fluorescence was restored. However, both designs require the continuous optimization of the aptamer binding position. In Walter et al.’s scheme [62], if the aptamer is too close to the pivot, the DNA origami arms may move together even in the absence of ATP, inducing RET. Thus, the region farthest from the pivot proved to be the optimal location for the aptamers. Conversely, in Xiong’s design, if the aptamer is too far from the central pivot of the DNA tweezers, background fluorescence leakage can occur due to the excessively short hybridization length.

Aptasensors may exhibit the same conformation as that upon target binding due to intramolecular remodeling, resulting in false positive signals, and this instability is difficult to control in solution [64]. Palma et al. solved this problem by organizing the cortisol aptamer with the single-molecule level using triangular DNA origami immobilized on a glass cover slide and adding the excess quencher strand to the solution phase side [64]. They proposed that the ability to control the surface density of allosteric molecules on DNA origami, in combination with reaction equilibrium theory, is crucial for preventing signal leakage from a single allosteric sensor. This approach also demonstrates applicability to any aptamer used in target-induced fit sensing.

The above studies indicate that when one end of the allosteric molecule is attached to the DNA origami surface, ligand binding events can drive conformational changes in the DNA origami. However, if researchers aim to reduce predefined sequence constraints (keeping the DNA origami staples independent of the aptamer), labeling the reporter group on the recognition element becomes necessary [62,64]. This proximity effect may interfere with molecular interactions and prevent the conformational signal from being transmitted remotely through the DNA origami, thereby affecting the sensitivity of the DNA origami biosensor. Designing spatially distinct recognition and signal transduction modules requires that the DNA origami carry specific staples complementary to the aptamers [63], but this can limit the universal applicability of the DNA origami biosensor. Ke et al. proposed a solution to this problem by introducing “corner lock strands” for rhomb-shaped DNA origami nanoactuators. Each “corner strand” at the top or bottom corners contained a segment of a 34-base single-stranded overhang that folded into a G-quadruplex in the presence of K^+^, thereby controlling the angle of the rhomb-shaped origami arms (Figure 6B) [43]. Since the recognition sequence for K^+^ was completely isolated, the DNA origami arms could be synthesized using constant sequences. The distance between different signal molecules on two DNA origami arms (on one side of the top or bottom corner) was controlled remotely by the joint motion caused by the corner lock opening and closing, allowing for the independent programming of the recognition module and the signal module. As the buffer was exchanged, the rhomb-shaped angle exhibited a “small-large-small” distribution, and the fluorescence correspondingly switched between “quenching-emission”, indicating that this origami could dynamically detect changes in buffer composition. Thus, completely orthogonal, highly flexible input (recognition) and output (signal) modules enable DNA origami biosensors to report low-concentration targets with high sensitivity while preserving the original structures carrying fluorescence reporters, making it easy to customize multiple allosteric molecule-driven DNA origami biosensors.

While just three examples cannot represent all cases, introducing allosteric molecules into DNA origami is relatively straightforward, which is why most researchers are interested in this approach. In most situations, molecular allosterism causes changes in the end-to-end distance of DNA origami, serving as a simple signaling mechanism. Additionally, the process requires only the direct connection of two well-understood components (DNA origami and allosteric molecules), without the need for designing additional complex behaviors such as DNA walkers, shape-encoded DNA LEGO, sequence-encoded TMSD, or force-driven DNA pistons.

#### 3.2.4. Switch Between Duplex and Triplex

**Figure 7:**
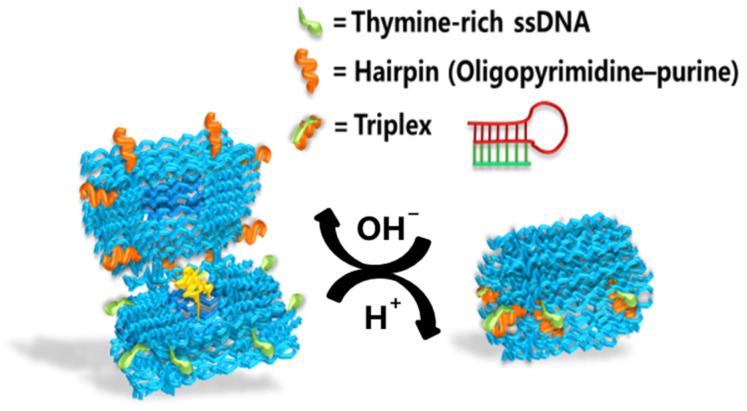
Sensing scheme based on DNA triplex forming and dissociation. The Hoogsteen-type triplexes can respond to pH changes and control the “on” and “off” of DNA origami, which is an ideal template for developing biosensors [66].

Previously, many scientists have observed that repeated pyrimidine–purine sequences in double-stranded DNA tend to bind a third strand at the major groove. If the third strand is rich in pyrimidine, it binds in a parallel direction to the central strand (polypurine strand), forming T-AT and C-GC triplets via Hoogsteen hydrogen bonding [65]. The protonation/deprotonation of the cytosine N_3_ atom controls the formation and dissociation of the DNA triplex, and positive ions can reduce the electrostatic repulsion between the three polyanionic strands, enhancing the stability of the DNA triplex [65]. This means that the Hoogsteen-type triplex can respond to pH changes. Ijäs et al. took advantage of this by designing latches for spherical DNA nanocapsules consisting of two half-capsules with a central cavity. On one half-capsule, eight 20 bp polyT-A hairpins were placed, and on the other half, a corresponding number of 20 nt polyT ssDNAs were fitted [66]. At pH 6.4–6.8, the polyT-A hairpins formed a triplex with the polyT ssDNA, causing the nanocapsule to close. However, when the pH rose to 7.6–8.0, the latch opened, and the nanocapsule exposed its inner cavity (Figure 7). By loading cargo, such as AuNPs, into the inner cavity, the nanocapsules could target cancerous areas for photothermal therapy, as cancer cells typically have a higher pH (7.3–7.6) than healthy cells. They also anchored HRP to the cavity, which is an enzyme ideal for catalyzing hydrogen peroxide in chromogenic reactions. Although they did not specifically propose a biosensor, a DNA origami colorimetric biosensor based on HRP is feasible due to its visual properties.

#### 3.2.5. Shape Transformation Caused by Multidentate Binding

**Figure 8:**
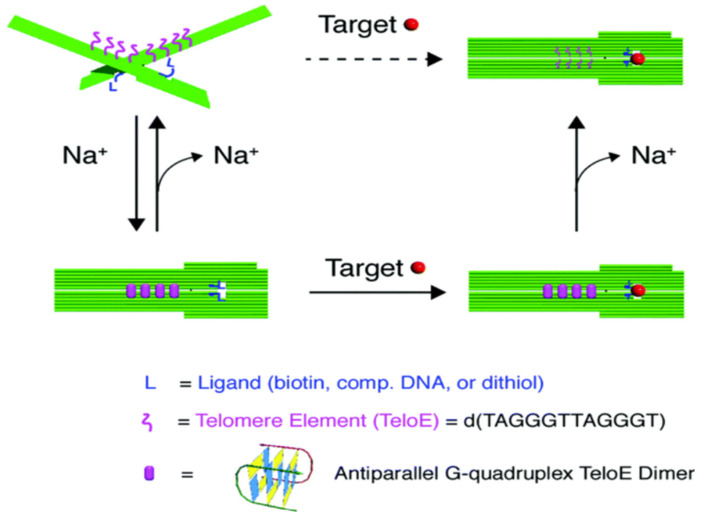
Sensing scheme dominated by ligand–target multidentate binding [68]. DNA origami can open or close to indicate ligand–target binding without signal transduction capabilities. Copyright 2017 Chemical Communications.

Except for some allosteric elements that can transmit their own structural changes to DNA origami, many recognition elements only undergo multidentate binding, such as streptavidin–biotin interactions, and the anchoring to AuNPs by thiolated molecules, which cannot indicate binding events are often used in surface modifications [64,67]. However, DNA origami allows multidentate binding to be transformed into a visible output, enabling the biosensing of targets of interest. Kuzuya et al. reported a sensor composed of two ligand sets that independently regulated the structure switching of DNA origami pliers, achieving the sensitive detection of streptavidin and AuNPs (Figure 8) [68]. Biotin–triethylene glycol and DTPA-HEG (containing dithiol)-bearing staple strands were attached to each of the concavities forming the jaws, allowing streptavidin or AuNP in the solution to pinch the DNA pliers through multidentate binding interactions with the corresponding ligands. The researchers demonstrated that supplementing Na^+^-sensitive elements (such as telomeres, which can form antiparallel G-quadruplexes under Na^+^ induction) over the fulcrum increased the detection rate in the presence of streptavidin and significantly enhanced the signal-to-noise ratio corresponding to AuNPs, compared with sensors that placed multidentate binding ligands only at the jaws. The use of multidentate binding ligands also improved the sensitivity of the DNA origami pliers to Na^+^. While the current sensor reads signals through AFM, it could potentially be combined with fluorescent dyes and expanded to detect any combination of two substances for future applications.

## 4. Advantages and Current Limitations of DNA Origami Biosensors

DNA origami biosensors offer several advantages compared to conventional optical biosensors. Firstly, they can precisely control the distance between optical elements. As is widely known, fluorescence-quenching or resonance energy transfer mechanisms are highly sensitive to intermolecular distance, with quenching efficiency or energy transfer efficiency depending on the distance between the donor and acceptor [69]. To achieve a higher signal-to-noise ratio, it is crucial to maintain a greater distance between elements in the absence of the target and bring them as close as possible when binding events occur. DNA origami excels at this task. Secondly, sensor arrays can be organized within both two-dimensional and three-dimensional origami structures. DNA origami provides many more binding sites for reporters or recognition elements compared to conventional biosensors, such as antibody-based sensors, molecular beacons, or in vitro transcription circuits. This enables one target to trigger responses from dozens of fluorophores, effectively functioning as multiple single biosensors operating simultaneously in a confined area, which significantly improves sensitivity [32]. Thirdly, DNA origami enables measurements at the single-molecule level. In traditional biosensors, signals typically arise from the average response of a collective in the solution. In contrast, DNA origami offers a large sample volume (equivalent to hundreds of single-molecule detections) due to its single-molecule resolution, making highly accurate individual measurements possible [32]. Additionally, DNA origami biosensors possess superior biocompatibility and biodegradability and are nearly non-toxic [70,71], making them particularly well-suited for use in living organisms. Finally, DNA origami biosensors are likely more stable than protein-based biosensors (e.g., immunosensors) and small nucleic acid-based biosensors [72]. This is because DNA duplexes embedded within larger DNA nanostructures exhibit greater resistance to nuclease degradation compared to free DNA duplexes [72]. The large size of these structures generates steric hindrance, preventing nucleases from effectively binding to the DNA [73]. Furthermore, highly rigid DNA within larger nanostructures has a lower binding affinity for nucleases [74], and high DNA and charge density may also inhibit nuclease interactions with the DNA [75]. In addition, most DNA termini are shielded from protease attacks [76].

However, there have been just over 100 reports of DNA origami sensors up to now, compared with more than 400 cases of sensors developed by CRISPR technology that were proposed nearly in the same period [77,78]. Except for DNA origami sensors’ construction strategy, which has not been fully explored, their manufacturing process and application performance are also an important reason hindering the popularization of DNA origami biosensors. On the one hand, precisely customized DNA origami requires dozens to hundreds of short staples, a considerable number of which are non-covalent or covalent-modified, making the production cost unacceptable and increasing the risk of synthetic failure [79]. In this case, people may prefer to use simple DNA sensors (e.g., CRISPR-Cas [80], DNA tweezers [81]). From the perspective of functionalization, given orthogonal reporting groups, the same covalent modification strategy allows each small nucleic acid a unique sensor for integrated use [82], but the same covalent modification strategy causes the random modification of orthogonal reporting groups on DNA origami, breaking the uniform standard for multiplexing. Essentially speaking, if researchers use non-covalent modifications relying on DNA-specific hybridization, they still synthesize heterogeneous ssDNA-X (X represents quantum dot, peptide, dye, metal nanoparticle, etc.), which conjugates first [83], where ssDNA-X conjugates could be directly applied as probes for imaging or sensing [84] without requiring the use of DNA origami. Due to the reversible nature of DNA hybridization and the inherent nanoscale addressability, short staples may not assemble with 100% accuracy at the intended positions, and there is a lack of effective methods to monitor the origami assembly process [85,86]. Consequently, it is difficult to separate fully functionalized DNA origami from partially functionalized constructs, as current purification methods are insufficient for this task [86,87]. Furthermore, the growing diversity of functional groups and the emergence of complex three-dimensional structures make it increasingly challenging to efficiently and accurately characterize DNA origami using atomic force microscopy (AFM) and transmission electron microscopy (TEM) [86]. Methods based on spectroscopy or the nanostructure’s physical separation are promising characterization strategies in the future [86].

On the other hand, existing origami sensors work well in buffer systems, but their performance in complicated matrices is rarely reported, and they are incapable of providing a useful reference for clinical translation and real-world scenarios. In addition to the plasmonic enhancement and multi-fluorophore array [32,58], the signal improvement mechanisms for origami sensors based on nucleic acid amplification circuits have not been specifically reported, which is expected because of nucleic acid species’ compatibility. Amplified signals could improve the limit of detection for origami sensors to the subpicomolar level, which would be beneficial for uncovering new disease biomarkers [88].

## 5. Conclusions and Outlook

DNA origami biosensors developed in the past decade can be reasonably divided into two groups based on whether the target binding event follows the “competition principle”. General guidelines for designing origami sensors within each group have been proposed. We observed that strategies other than base pairing are suitable for non-nucleic acid targets, including biomarkers, ions, small molecular contaminants, ATP, and others. It is evident that DNA origami biosensors offer significant advantages over traditional single-molecule sensors, although challenges remain in achieving high-purity functionalized origami and effectively characterizing them.

Moving beyond proof-of-concept to practical applications is particularly important for advancing DNA origami sensing schemes in engineering. The liquid phase environment poses challenges for transporting and preserving DNA origami biosensors, so finding suitable carriers could accelerate their commercialization. For example, microfluidic paper-based analytical devices (PADs) have demonstrated compatibility with simple DNA nanostructures, where nucleic acid reactions are initiated through hydration activation, and sample processing is sequential and automatic [89,90,91]. This approach can simplify the cumbersome procedure of multi-step reagent manipulation in laboratory settings. Additionally, some nucleic acid circuits have been incorporated into hydrogels to develop optical strain sensors, which can monitor human health due to the excellent elasticity and biocompatibility of hydrogels [89,90]. When integrated with such carriers, DNA origami can fully utilize its unique advantages.

DNA origami, consisting of DNA building blocks with complementary shapes, can theoretically be assembled infinitely, and by defining surface protrusions and indentations, it can form any desired structure. If researchers combine fluorescent dyes with selected duplexes within building blocks through the base intercalation or groove chimerism, it becomes easy to organize “custom patterns”, similar to the site-specific labeling of quantum dots on the DNA origami surface [92]. The large 2D plane formed in this way may exhibit properties similar to LED screens. Moreover, due to the high biocompatibility of DNA, this supramolecular fluorescent material is expected to become an important substrate for wearable monitoring devices and electronic skin.

It is essential to enhance the focus on producing DNA origami-based Surface-Enhanced Raman Scattering (SERS) sensors for clinical diagnosis and quality control. Since the development of SERS, researchers have been primarily concerned with improving the specificity of low-concentration analyses, achieving tunable sensitivity, and reducing the heterogeneity of detection substrates. The plasma fields created by traditional lithography techniques are not ideal, as the size, shape, and number of nanoparticles that form these fields are often uncontrolled, significantly affecting the accuracy and consistency of the results. DNA origami offers a bottom-up approach to fabricating plasma hotspots, where the distance between metal nanoparticles and their orientation can be continuously adjusted until the configuration with the highest enhancement factor is achieved. Additionally, it allows for the cascading of different fields [93,94]. More importantly, DNA origami can fix the target at a designated position within the plasma field, enabling specific analyses at the single-molecule level. These advantages address the shortcomings of traditional SERS detection and pave the way for a new platform suitable for large-scale applications. Currently, researchers in the field of DNA origami-based SERS sensors remain focused on Raman enhancement results and mechanisms, often using model analytes (such as fluorescent dyes or proteins) to examine the signal. Before single-molecule detection, analytes must be labeled in advance [95]. However, the matrix’s effects in real-world samples (such as milk or lake water) are highly complex, making it impractical to label the targets beforehand, and ensuring that targets uniformly fall within the Raman enhancement region is challenging [20,22]. Designing application-specific detection schemes and enabling multi-target parallel analysis are critical bottlenecks that need to be addressed to advance the industrialization of DNA origami-based SERS sensors.

Inspired by natural photosynthesis, using DNA origami to create biomimetic light-capturing systems could facilitate the development of novel optoelectronic devices and nanoreactors. DNA origami biosensors play an important role in these systems because natural light capture is a multi-step sequential energy transfer process [96]. A similar tricolor FRET light-cascade DNA origami biosensor has been reported, which responds to monovalent cations such as Na^+^ or K^+^ [97]. Wang et al. developed a stable white light-emitting supramolecular vesicle material that converted captured solar energy into chemical energy, catalyzing the dehalogenation of α-bromoacetophenone in water. However, they did not explore its further application in optical materials, and the system lacked the ability to respond to specific targets [96]. Combining their approach with DNA, origami biosensors could be an interesting form of direction, as current DNA origami biosensors do not have the capability to further transform and utilize light signals for processes such as photocatalytic oxidation or photoelectric conversion. Additionally, DNA origami biosensors could provide the aforementioned white light-emitting supramolecules with the ability to interact with the environment. Light-capturing systems derived from DNA origami biosensors may, thus, exhibit more advanced functionality.

There is also significant potential for DNA origami to explore environmental monitoring applications. It is encouraging to see the development of pH-sensitive origami biosensors, but there have been few attempts to apply them to water pollution detection. Researchers could test their specificity and sensitivity in simulated wastewater and then compare their performance with gold standards like pH meters through spike and recovery experiments. Ultimately, they could be used to assess the acidification or salinization of real environmental samples, such as soil solutions. Moreover, the recognition capability and binding mechanisms of DNA to organic contaminants and inorganic acids remain largely unexplored. Some previous studies have shown that aromatic ring structures within contaminants can interact with ssDNA [98], often quenching the fluorescence from quantum dots [99]. This suggests that quantum dot-modified DNA origami could be developed into biosensors with broad-spectrum identification abilities for aromatic pollutants. However, specificity is considered more crucial when developing biosensors. Thus, the key challenge in designing DNA origami biosensors sensitive to pollution lies in establishing a relationship between the origami structure and specific pollutants. Given the customizable nature of aptamers and their compatibility with DNA origami, it is foreseeable that more pollutant-specific DNA origami biosensors will be developed and put to use in field applications.

## Data Availability

Data are available upon request from the authors.

## Abbreviations

NTA-Ni	Nitrilotriacetic acid–nickel ions
Sulfo-SMC	Sulfosuccinimidyl 4-[N-maleimidomethyl] cyclohexane-1-carboxylate)
ELISA	Enzyme-linked immunosorbent assay
6-FAM	6-carboxyfluorescein
BHQ-1	Black hole quencher 1
Popo_3_	Dimeric cyanine nucleic acid stains
ADP	Adenosine-diphosphate
ATP	Adenosine-triphosphate
ATPase	A class of enzymes that catalyze the decomposition of ATP, producing ADP and free phosphate ions or which catalyze its reverse reactions
PEG	Polyethylene glycol
ΔΔGPEG	The change in free energy difference
eGFP	Enhanced green fluorescent protein
HRP	Horseradish peroxidase
LED	Light-emitting diode
